# The influence of mature oak stands and spruce plantations on soil-dwelling click beetles in lowland plantation forests

**DOI:** 10.7717/peerj.1568

**Published:** 2016-01-12

**Authors:** Tereza Loskotová, Jakub Horák

**Affiliations:** Department of Forest Protection and Entomology, Czech University of Life Sciences Prague, Prague, Czech Republic

**Keywords:** *Athous*, Bioturbation, Sessile oak (*Quercus petraea*), Spatial partitioning, Patch level, Elateridae, Norway spruce (*Picea abies*)

## Abstract

Most European forests have been converted into forest plantations that are managed for timber production. The main goal of this paper was to determine the difference between mature native sessile oak (*Quercus petraea*) stands and non-indigenous Norway spruce (*Picea abies*) plantations, with respect to communities of *Athous* click beetles in approximately 6,500 ha of lowland plantation forest area in the Czech Republic. *Athous subfuscus* was the most abundant and widespread species, followed by *A. zebei* and* A.* *haemorrhoidalis*, while *A. vittatus* was considered rare. Spatial analysis of environmental variables inside studied patches showed that the species composition of *Athous* beetles best responded to a 20 m radius surrounding traps. The species’ responses to the environment showed that *A. vittatus* and *A. haemorrhoidalis* preferred oak stands, while *A. zebei* and *A. subfuscus* were associated with spruce plantations. In addition, oak stands showed higher diversity of beetle communities. The studied species are important for their ecosystem services (e.g. predation on pests or bioturbation) and seem to tolerate certain degrees of human disturbances, which is especially beneficial for forest plantations managed for timber production.

## Introduction

Forests are biologically diverse ecosystems, representing some of the richest biological areas on Earth (Lindenmayer et al., 2002; Wesolowski, 2005). While many species thrive, some forest organisms are threatened as a result of deforestation, fragmentation, change in tree species composition, climate change and other stressors like fire suppression (Carnus et al., 2006).

Semi-natural forests are rare in Europe (Wesolowski, 2005). Most forests have been cleared and converted into agricultural land or into regularly cut forest plantations (Horák, Chobot & Horakova, 2012). Many of the broadleaved forests of lowland Europe were replaced by coniferous stands (Carnus et al., 2006). Large-scale intensive forestry has led to a shift in the quality of forest habitats, which has influenced the diversity of forest organisms (Brockerhoff et al., 2008). However, managed forests can still have a high ecological value (Bauhus, van der Meer & Kanninen, 2010) particularly compared to intensively managed agriculture land.

The distribution of forest organisms in fragmented landscapes is influenced by structural characteristics of the forest, such as patch quality, configuration or history (Collinge, 2009; Horák & Rebl, 2013). While quality and the spatial aspects of forest fragmentation (i.e. isolation) have received much attention recently (e.g. Mason & Zapponi, 2015), the temporal dimension of habitat fragmentation (Brunet, 1993; Horák, Chobot & Horakova, 2012)–e.g. through the long-term dominance of native tree species (Carnus et al., 2006)–has been less often the focus of attention.

The occurrence of many forest organisms is assumed to be exclusively or largely restricted to forests with geographic habitat continuity–e.g. the presence of matured and over-matured native broadleaved tree stands in lowlands (Peterken, 1974; Brunet, 1993), while some other species have good dispersal abilities and are able to spread together within forests of suitable tree composition (e.g. Norway spruce associates) when they are planting or spreading (Roder et al., 2010).

The click beetles, Elateridae, are one of the most ecologically diverse families of beetles (Leseigneur, 1972; Laibner, 2000; Johnson, 2002). Adults are active in the afternoon and evening and some can be effectively collected using window traps (Horák & Rebl, 2013). Click beetles from the genus *Athous* Eschscholtz are known for their beneficial function of bioturbation and predation on the larvae of Hymenopteran and Lepidopteran pests (Laibner, 2000). The adults occasionally feed on buds, leaves and below-ground parts of crop (Douglas, 2011), although the damage is insignificant in central Europe (Laibner, 2000). Most observation records of adults are from herbs, shrubs or lower branches, and the development of larvae takes years (Laibner, 2000; Johnson, 2002).

Recently, five *Athous* species have been reported from the Czech Republic (Dusanek & Mertlik, 2012)–namely, *Athous haemorrhoidalis* (Fabricius, 1801), *A. subfuscus* (Müller, 1767), *A. vittatus* (Fabricius, 1792), *A. zebei* (Bach, 1854) and *A. bicolor* (Goeze, 1777). *Athous haemorrhoidalis* is widely distributed from lowlands to mountains, preferring open park landscapes, abandoned agricultural and urban areas, and grasslands (Laibner, 2000). *Athous subfuscus* is widely distributed in all types of forests, especially in clear-cuts and adjacent sites (Laibner, 2000). *Athous vittatus* and *A. bicolor* are indicated to prefer close to natural open canopy broadleaved woodlands with lower altitudes and *A. zebei* is indicated to prefer coniferous woodlands at higher altitudes (Laibner, 2000). *Athous haemorrhoidalis* and *A. vittatus* both have larvae that dwell in the sun-warmed soils and feed on dead invertebrates. *Athous subfuscus* and *A. zebei* larvae are predaceous (Laibner, 2000). They dwell in the soils and litter of shaded woodlands, especially under mosses. The fifth species distributed in the Czech Republic, *A. bicolor*, is indicated to be relatively widespread (Laibner, 2000), although to our knowledge, the trapping success of this species is very low, with the species only having been trapped in semi-natural woodlands (Horák & Rebl, 2013). Its ecological requirements are quite similar to those of *A. vittatus* (Laibner, 2000).

### Aims

Our prediction was that stands with planted non-indigenous trees influence the *Athous* click beetles in lowland plantation forest areas due to their known habitat requirements. Thus, the general goal of this study was to explore the influence of mature native sessile oak (*Quercus petraea*) stands and non-indigenous Norway spruce (*Picea abies*) plantations on studied click beetles. More specifically, we explored: (i) the response of the studied community to the forest environment at the most suitable patch level through spatial partitioning and (ii) the individual *Athous* species’ responses to the environment.

## Material and Methods

### Study group

The genus *Athous* is poorly studied group of click beetles (Roberts, 1919; Wolters, 1989). In spite of this fact, they are mentioned as both potential pests (feeding on buds and leaves) and as beneficial organisms (predation and bioturbation) (Laibner, 2000). They, furthermore, could be the dominant part of the community of soil-dwelling organisms in forests. Their study could bring us important information on how forest organisms could be affected by alteration of tree species composition–mainly due to changes in vegetation structure caused by different litter decomposition effects of conifer vs. broadleaved and native vs. non-indigenous trees. Regarding their response to the forest environment, the species composition and individual species population densities of genus *Athous* were studied as dependent variables.

### Study area

**Figure 1:**
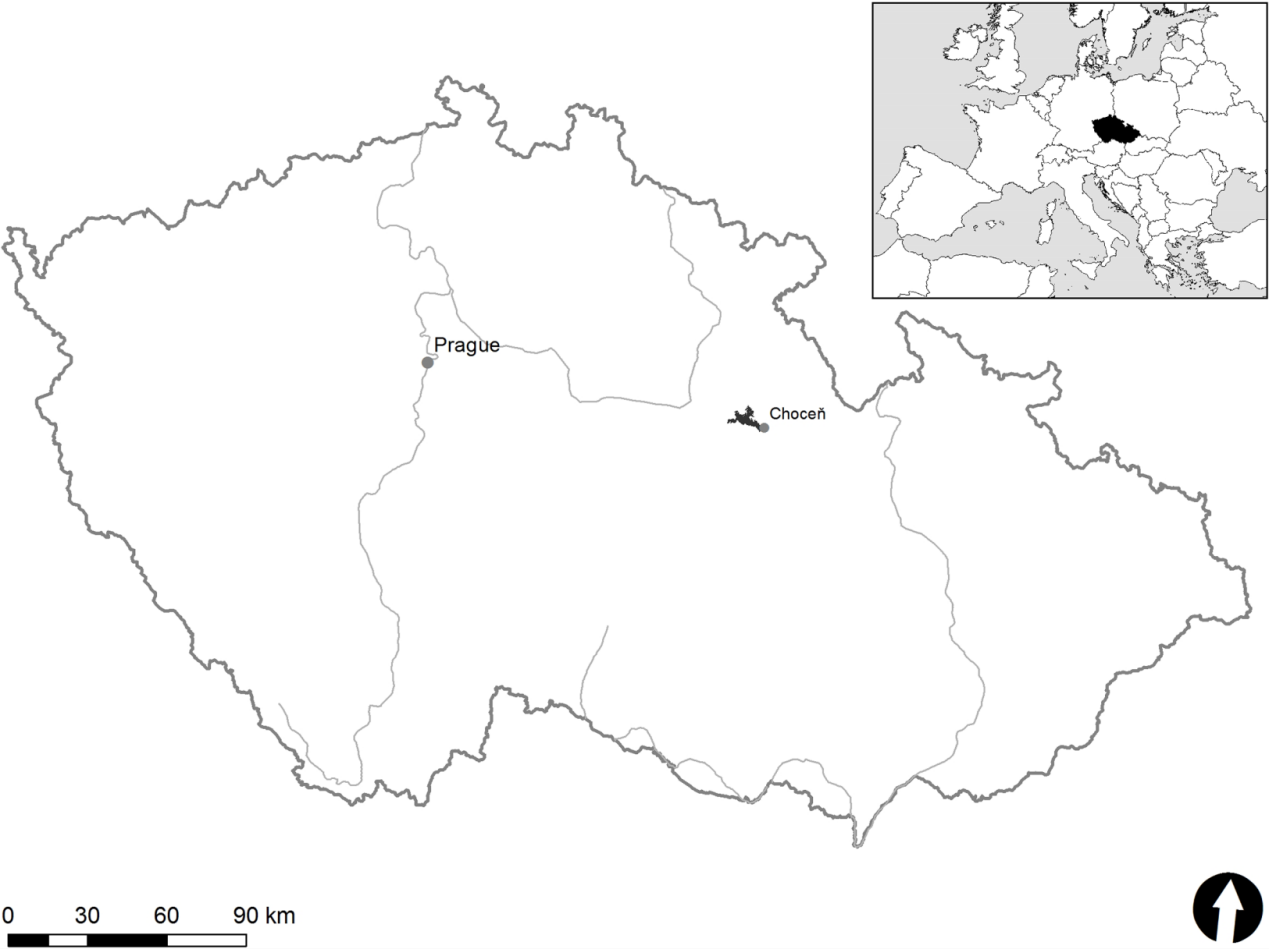
Location map of study area (black) near Choceň town in the Czech Republic.

The study woodland area of nearly 6,500 ha was situated in a spatially continuous area of the east-Bohemian woodlands, between the towns of Choceň and Holice (Pardubice Region, Czech Republic; Fig. 1). According to Neuhauselova (2001), a potential vegetation of forests in the area consists of oak-hornbeam forests mixed with European beech (*Fagus sylvatica*) forests with a scattered distribution of silver fir (*Abies alba*) and some oak forests with Scots pine (*Pinus sylvestris*). The historical distribution and abundance of Scots pine is unclear, although it has been recently found to dominate the area due to its commercial value (e.g. Cienciala et al., 2006). The former natural distribution of European beech in the area is uncertain and today, this tree is restricted to the slopes close to the Tichá Orlice River in the east. The European hornbeam (*Carpinus betulus*) is presently the only admixed tree species. Of the broadleaved trees, only sessile oak (*Quercus petraea*) now covers relatively large areas. A large number of forest stands have been planted over more than two centuries using non-indigenous Norway spruce (*Picea abies*).

### Site selection

Mature stands (i.e. more than 80 years old) dominated by sessile oak or Norway spruce that had more than 1 ha in total area were studied over the whole study area. Sessile oak and Norway spruce dominated stands were chosen as they best reflect the recent environmental condition of the forest with respect to its tree species composition in the past. In our study, oak stands represented former continuous vegetation and spruce plantations indicated spatial and temporal discontinuity. Due to possible significant influence of spatial autocorrelation and the effect of tourist beetles from non-forest and highly disturbed areas, their choice was limited to the two another parameters: (i) the minimal distance between sampling points in oak and spruce dominated stands, which was set to 50 m, (ii) as well as the distance to the woodland edge and/or to clear cut, which was also set to 50 m. This selection enabled us to sample in the 30 sites–i.e. 15 pairs of oak and spruce stands.

### Trap description

**Figure 2:**
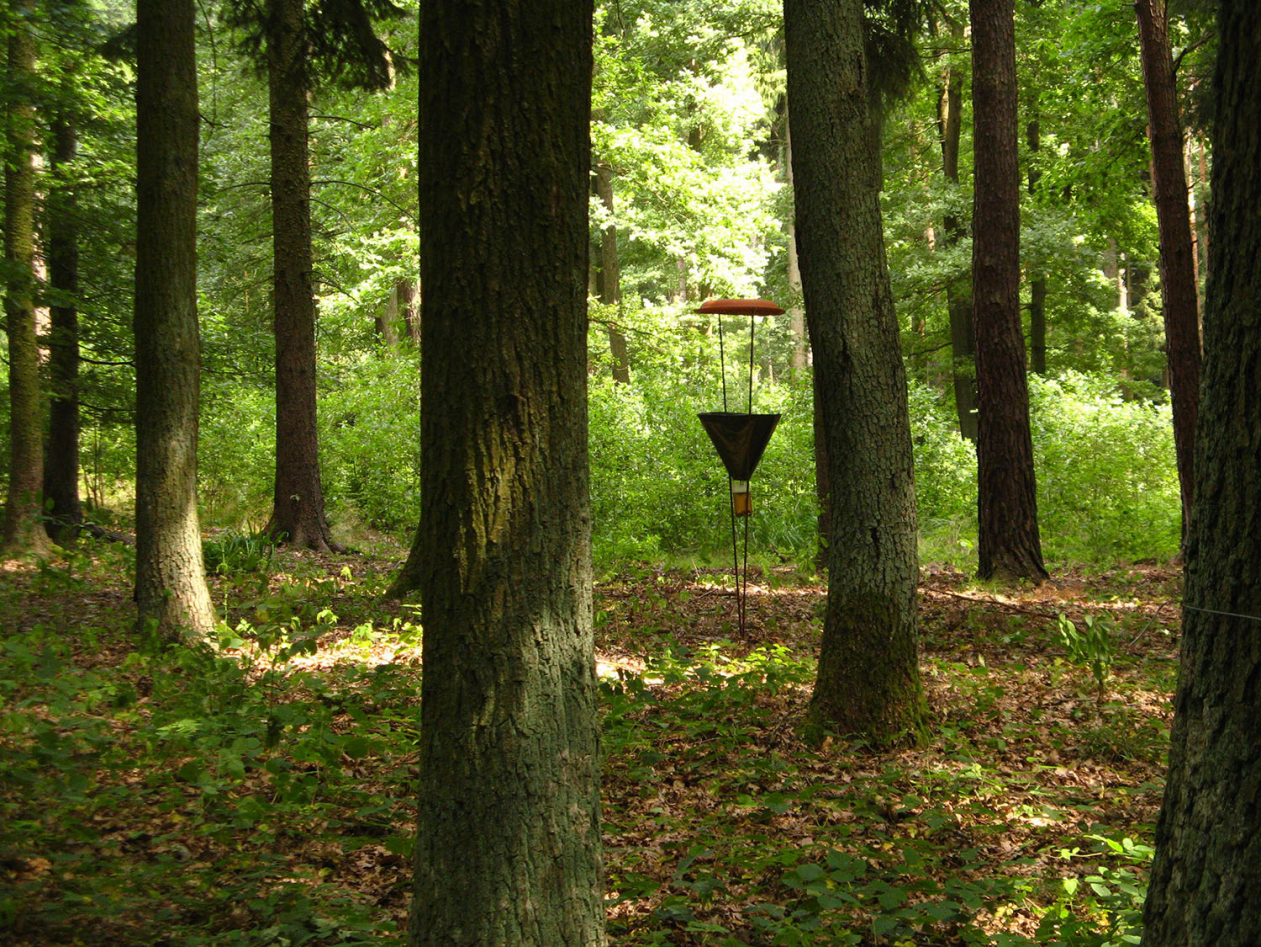
A window trap used to capture *Athous* click beetles (Elateridae) in lowland plantation forest area.

Crossed-panel window traps were used for this study. We installed one trap per site. Each trap consisted of three transparent plastic panes (one pane 0.4 × 0.5 m and two panes 0.2 × 0.5 m), a protective top cover (diameter 0.45 m), and a funnel leading down into a container holding a solution of water and salt with a small amount of detergent to reduce the surface tension of the liquid. This solution preserved the insects but did not attract them (Horák, 2011). The height of the center of the trap was approximately 1.3 meters. Traps were fixed using two iron sticks on two opposite sides and they were positioned at the centers of the stands (Fig. 2). All of the traps were activated at the beginning of March and deactivated at the end of September, 2011, resulting in eight sampling efforts (25.3., 25.4., 20.5., 10.6., 5.7., 30.7., 25.8. and 20.9.). Thus, each trap was working for a period of 179 days (i.e., 5,370 days for our trapping design). We assumed that every individual had an equal probability of being captured.

### Environmental variables

The main focus was on environmental variables (as independent variables) at the circular patch scales surrounding each trap, which potentially best described requirements of the studied group within the studied plantation forests (Table 1). All of the studied variables, except for canopy openness (measured in the viewing angle of 180°), were measured as a percentage of coverage of a circle with a radius of 10 meters (314 m^2^) and then in those with twice the radius of the previous samples–i.e. 20 (1,256 m^2^) and 40 (5,024 m^2^) meters (Table 1).

Canopy openness, as an expression of the light conditions of the study site, was measured using a Nikon COOLPIX 995 camera with a Nikon FC-E8 Fisheye converter. Each photograph was taken at the top of the trap, approximately 1.55 m above ground. All photographs were then evaluated using Gap Light Analyzer 2.0.

Total representation (i.e., % of tree species in the patch) of mature sessile oak in the tree species composition of the overstory was measured as a reflection of the maintenance of the former vegetation. The representation of mature Norway spruce was measured as a reflection of the historical anthropogenic disturbance of the stand. The representation of other deciduous and coniferous trees was also measured. The conditions in the understory were measured, with focus placed on the total coverage of shrubby vegetation, vascular plants, bare soil and mosses (Table 1).

**Table 1:** Range of habitat variable variation between sites at the lowland plantation forest area. Descriptive statistics of percentage values of the studied variables are shown. Canopy openness represents openness of tree canopy cover above every trap. Oak and spruce represent their percentage of tree species composition. Plants, mosses and shrubs represent percentage of cover at the understory level. Except for canopy openness, all other variables were measured at patches of 10, 20 and 40 m radius surrounding the traps. Other coniferous and deciduous trees and bare soil were excluded because of multi-collinearity.

Predictor	Radius (m)	Mean ± S.E. (%)	Min-Max (%)
Canopy openness		9.44 ± 0.36	6.74–14.46
Oak	10	44.60 ± 8.22	0–100
Spruce		42.17 ± 7.99	0–100
Plants		44.60 ± 7.23	0–100
Mosses		8.67 ± 3.22	0–60
Shrubs		4.10 ± 1.99	0–45
Oak	20	43.23 ± 7.56	0–95
Spruce		38.87 ± 7.07	0–100
Plants		47.07 ± 6.39	0–100
Mosses		8.10 ± 2.95	0–60
Shrubs		5.13 ± 1.89	0–40
Oak	40	38.77 ± 6.58	0–95
Spruce		38.83 ± 5.98	0–90
Plants		48.30 ± 5.61	10–100
Mosses		9.47 ± 3.37	0–75
Shrubs		5.73 ± 1.90	0–40

### Statistical analyses

Due to limited number of traps used (based on criteria mentioned in the Site selection section), sufficiency of number of traps used for statistical power to detect an effect (i.e. trapping success) was assessed using EstimateS 8.2. Sample-based rarefaction (Mao Tau function with 95% confidence intervals) and the Chao estimation functions were computed, with the number of randomizations set at 1,000.

Principal components analysis (PCA) of species composition of the study group of *Athous* click beetles regarding the site character was computed in CANOCO 4.5 (ter Braak & Smilauer, 2002) for the analysis of discrimination between the samples, and then was visualized in CanoDraw 4.14 (ter Braak & Smilauer, 2002).

Redundancy analysis (RDA) of species composition, as a dependent variable, was computed with 9,999 unrestricted permutations under the full model in CANOCO. All environmental variables with a variance inflation factor (VIF) higher than ten were first excluded from the final analyses due to multi-collinearity (Table 1). Control for possible influence of spatial autocorrelation was included as the co-variable–i.e. coordinates and their crossed and square products (x, y, xy, x^2^, y^2^; e.g. Horák, 2013). Spatial partitioning of the studied patches helped with the selection of the best spatial extent (i.e. radii of 10, 20 or 40 m) of the analyses of response of the species composition of *Athous* click beetles to the studied environmental variables (Horák et al., 2013). The final choice of patch space for analyses was based on the highest variance explained by canonical axes, as derived from RDA.

Individual species’ response to the environmental variables at the previously selected most suitable patch area was computed in the same way, as previously described with regards to RDA, and was visualized in CanoDraw using species-environmental and Shannon diversity-based data attribute-environmental biplots. The variance explained by the studied environmental variables and its significance was computed in CANOCO with 9,999 unrestricted permutations under the full model.

Generalized linear models (GLM) were computed in CanoDraw, with model selection based on Akaike information criterion (AIC) statistics and with Gaussian distribution for response of total species composition to individual environmental variables, while Poisson distribution was used for individual species data in the same way.

## Results

Four of the five *Athous* species reported from the Czech Republic (Dusanek & Mertlik, 2012) were trapped. *Athous subfuscus* was the most abundant and widespread species, followed by *A. zebei* and *A. haemorrhoidalis*, while *A. vittatus* was collected rarely, at only five sites (Table 2).

**Table 2:** Presence and abundance of *Athous* species at study sites. Descriptive statistics of the studied species trapping success, their abundance and species richness in lowland plantation forest area.

Name	Sites	Individuals	Mean ± S.E.	Min-Max
*A. haemorrhoidalis*	22	194	7.07 ± 1.73	0–42
*A. subfuscus*	30	942	35.60 ± 4.62	2–110
*A. vittatus*	5	21	0.70 ± 0.43	0–12
*A. zebei*	25	248	8.57 ± 1.60	0–29
Individuals/site			51.93 ± 4.90	6–126
Species/site			2.70 ± 0.11	2–4

The use of thirty traps in our study was enough, and the use of twenty traps was found to be sufficient in similar studies–namely, observed species richness reached the asymptote at 19 traps (±1 C.I. 95%). Chao 1 and Chao 2 estimators indicated sufficiency at 17 traps.

**Figure 3:**
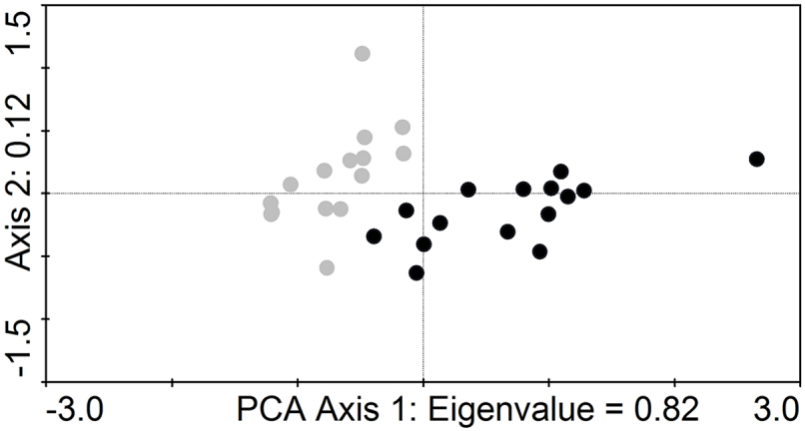
Sample based scatter-plot of species composition of soil-dwelling click beetles. Results as derived from principal components analysis (PCA) illustrating the discrimination between the samples in stands dominated by sessile oak (grey dots) and Norway spruce plantations (black dots) in the lowland plantation forest area.

There was a difference between the beetle communities of oak- and spruce-dominated stands. It explained nearly 82% of variance of the data in PCA, with only three sites overlapping on the first axis (Fig. 3).

**Figure 4:**
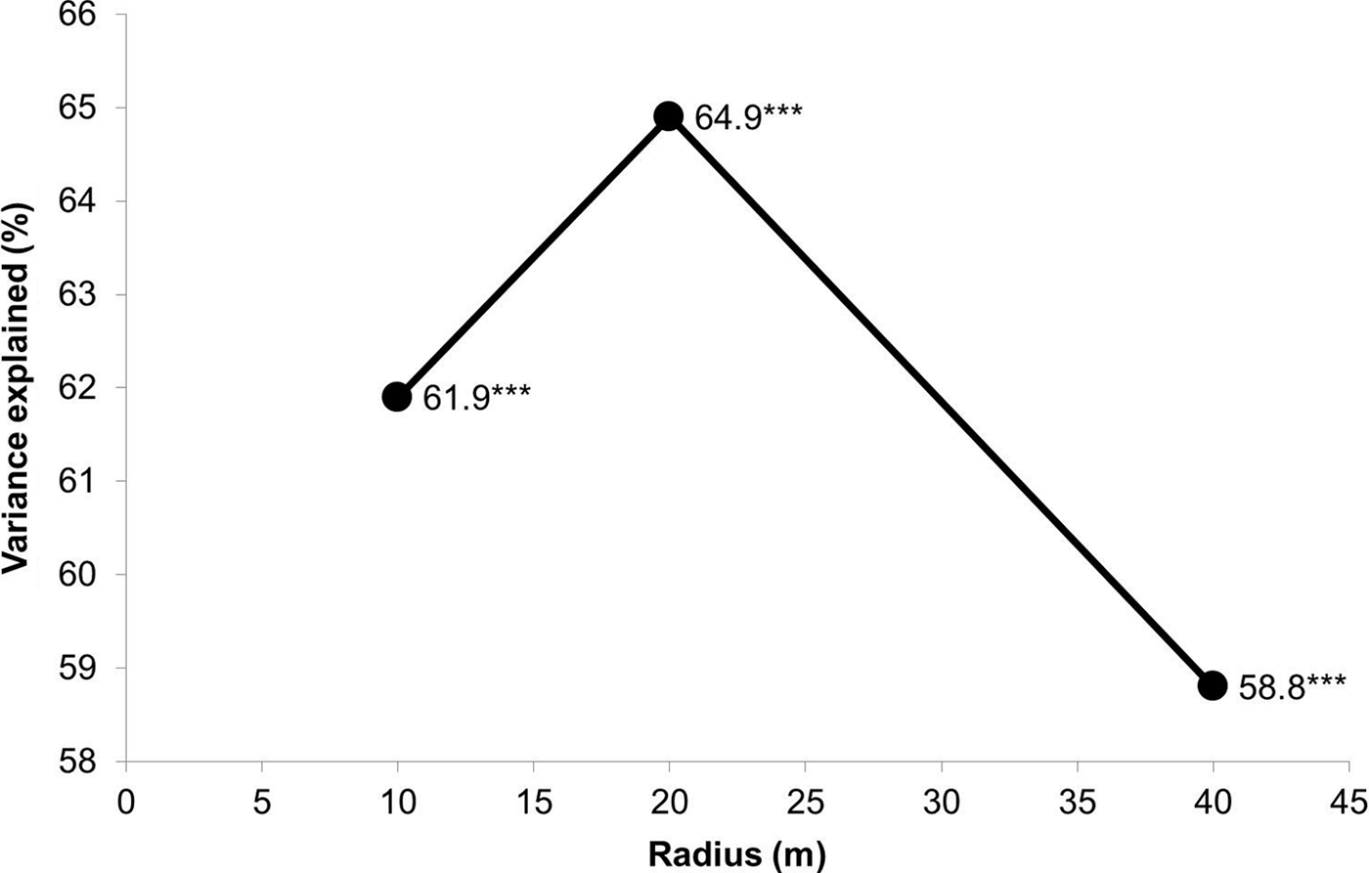
Results of spatial partitioning using variance explained by all canonical axes as derived from redundancy analyses (RDA). Species composition of *Athous* click beetles was dependent variable, and environmental independent variables were analyzed in a particular radius of surrounding patch in the lowland plantation forest area. Spatial terms (x, y, xy, x^2^ and y^2^) were included as co-predictors (*** is for P < 0.001).

The response of species composition to the environmental variables was the best at a 20 meters radius (1,256 m^2^ area) of the surrounding forest patch (Fig. 4). All axes for the 20 m radius together explained nearly 65% of the data variance. The worst response was at the longest radius of 40 meters, which did not exceed 60% of the explained variance. This also indicated that a selected distance of 50 meters (see the Material and Methods section) for possible overlapping among traps was sufficient.

**Figure 5:**
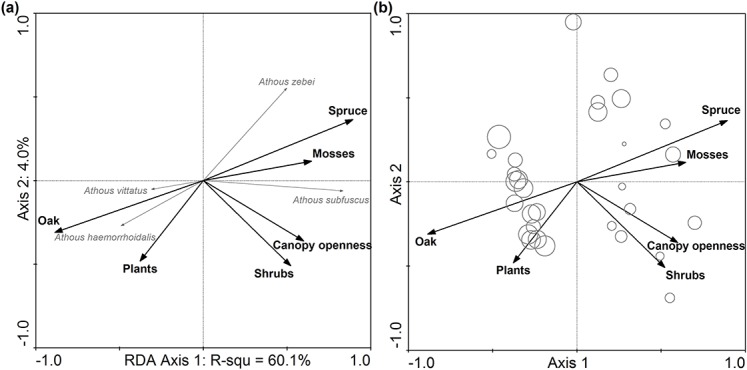
(A) Species-environmental biplot at 20 meters radius as derived from redundancy analyses (RDA) on species composition of soil-dwelling click beetles showing the response of species to environmental variables of the forest patch in the lowland plantation forest area. (B) Shannon diversity based data attribute-environmental biplot showing the diversity of samples. Note that in (A), the response of the species corresponded to the right angle projection of the end of the species arrow to the arrow of the particular studied predictor. The variance explained by each environmental variable and its *P* values is in Table 3. The size of the bubbles in (B) corresponds to the diversity of particular sample.

Species responses to the environmental variables at a 20-meter radius (Fig. 5A) showed that there were two species groups that were clearly discriminated on the first axis of RDA. *Athous vittatus* and *A. haemorrhoidalis* were on the left side of the diagram, preferring oak stands, while *A. zebei* and *A. subfuscus* were distributed on the right side of the biplot, with association to the spruce plantations. Samples in oak-dominated stands showed higher diversity (Fig. 5B). This is also illustrated by the negative *t* value of the first axis (*t* = −0.41), derived from significant GLM (*F* = 7.06; *P* < 0.01).

In relation to the studied beetle species composition, the percentage of spruce and oak in tree species composition at a radius of 20 meters surrounding the traps was significant and revealed the highest shared variance. From the variables at the understory level, coverage of mosses, together with shrubs, influenced the composition of studied beetles much more than did the coverage of plants, which showed an effect that was the lowest regarding the shared explained variance (Table 3). The shared variance explained by canopy openness with respect to the species composition of *Athous* click beetles was significant and was close to 25% (Table 3).

**Table 3:** Results of the studied species composition response to the forest patch predictors at a 20-meter radius in the redundancy analysis (RDA), explained in the lowland plantation forest area, sorted by percentage of explained variance. Note that significant *P* values are in bold. Canopy openness represents openness of tree canopy cover above every trap. Oak and spruce represent their percentage of tree species composition. Plants, mosses and shrubs represent percentage of cover at the understory level.

Predictor	Shared variance explained (%)	*F*	*P*
Spruce	50.0	29.04	**<0.001**
Oak	49.0	27.82	**<0.001**
Mosses	27.4	10.13	**<0.001**
Shrubs	24.9	8.84	**<0.01**
Canopy openness	24.1	8.45	**<0.05**
Plants	12.8	3.84	**<0.05**

Individual species’ responses (Table 4) showed that *A. haemorrhoidalis* and *A. vittatus* were positively related to the higher percentage of oak in the tree species composition and were negatively related to spruce, while the response of *A. subfuscus* and *A. zebei* was the opposite. Both *A. subfuscus* and *A. vittatus* showed the peak in preference for the tree species composition, with approximately 70% of spruce and oak, respectively. With respect to canopy openness, *A. subfuscus* preferred sun-exposed sites, while *A. vittatus* showed an increase in abundance with the shading of habitats. *Athous subfuscus* responded significantly to the coverage of mosses with the peak occurrence of around 40%, while also responding to the increasing coverage of shrubs. *Athous subfuscus* and *A. zebei* were negatively affected by the increasing coverage of the herb layer and the response of *A. vittatus* was slightly positive.

**Table 4:** Individual *Athous* species responses to the studied environmental predictors at a 20-meter radius in the lowland plantation forest area using GLM with Poisson distribution and selection based on AIC. Note that significant *P* values are in bold. Canopy openness represents openness of the tree canopy cover above every trap. Oak and spruce represent their percentage of tree species composition. Plants, mosses and shrubs represent percentage of cover at the understory level.

Species	Predictor	GLM	Linear response	*F*	*P*	AIC
*A. haemorrhoidalis*	Oak	linear	+	30.37	**<0.001**	175.0
	Spruce	linear	−	21.82	**<0.001**	201.0
	Canopy openness	quadratic	−	2.21	0.13	330.7
	Mosses	linear	−	3.85	0.06	314.6
	Plants	null	no	–	–	–
	Shrubs	linear	−	4.13	0.05	311.7
*A. subfuscus*	Oak	quadratic	−	21.59	**<0.001**	267.4
	Spruce	quadratic	+	19.51	**<0.001**	278.9
	Canopy openness	linear	+	7.17	**<0.05**	487.4
	Mosses	quadratic	+	7.12	**<0.01**	433.9
	Plants	linear	−	4.85	**<0.05**	522.1
	Shrubs	linear	+	4.55	**<0.05**	526.8
*A. vittatus*	Oak	quadratic	+	14.08	**<0.001**	54.6
	Spruce	linear	−	10.24	**<0.01**	74.0
	Canopy openness	linear	−	7.62	**<0.05**	80.1
	Mosses	linear	−	3.08	0.09	98.0
	Plants	linear	+	8.29	**<0.01**	78.3
	Shrubs	quadratic	+	1.66	0.21	105.4
*A. zebei*	Oak	linear	−	17.79	**<0.001**	179.4
	Spruce	linear	+	24.38	**<0.001**	155.9
	Canopy openness	null	no	–	–	–
	Mosses	linear	+	3.07	0.09	268.6
	Plants	linear	−	15.65	**<0.001**	198.4
	Shrubs	null	no	–	–	–

## Discussion

Our results can be summarized that the studied click beetles best responded to the environment at the middle selected patch area–i.e. with a 20 meters radius, and that dominant tree species in the patch were the most important with regards to the discrimination of studied beetle communities.

Studied click beetles best responded to the environment at the middle selected distance of a 20-meter radius surrounding the sampling site. This indicates the scales at which beetles with similar requirements are searching for suitable habitats. Even though the adults of most *Athous* species are known to be good dispersers compared to other beetles (Laibner, 2000; Johnson, 2002), the studies on beetle dispersal abilities have indicated that most beetle flight events are over shorter distances than previously predicted (Drag et al., 2011), even in pest species (Mercader et al., 2009)–i.e., on average, to one hundred meters. Nevertheless, populations of several insect taxa are known to release macropterous or highly dispersive individuals during times of high population densities (Kocarek et al., 2013). Thus, this surprisingly (and most probably) illustrates a relatively sedentary response to the environment in *Athous* beetles regarding the response to the patch of 20 m radius.

The study species were relatively clearly discriminated with respect to their relationships with the dominant tree species. Two necrophagous species (*Athous haemorrhoidalis* and *A. vittatus*) preferred oak stands and avoided spruce plantations, while two predators (*A. subfuscus* and *A. zebei*) showed the opposite response. The presence of two predaceous species in Norway spruce plantations is thus important and beneficial from the management point of view because of the higher vulnerability to environmental disturbances and potentially higher pest densities in soil of plantation forests of non-indigenous trees. The results also indicate that Norway spruce, as an autochthonous tree for the mountainous areas of the Central Europe, is also able to promote its habitat associates in areas of lower altitudes, which has been recently indicated (Roder et al., 2010).

The results showed that most of the studied species are able to reach high levels of abundance in mature stands within the plantation forests. Only *A. vittatus* was rather rare and was most abundant in relatively artificially undisturbed and mostly over-matured oak-dominated stands (based on our observation). This species preferred stands where oak accounted for between 60–80% of the tree species composition and with higher coverage of the herb layer in the understory. *Athous vittatus* is also known to be associated with sun-exposed woodlands (Laibner, 2000), although the results showed a relatively surprising association with closed canopy stands.

A relatively high abundance of *A. zebei* may be considered surprising because this species is indicated as being to be associated with mountainous and partly sub-mountainous woodland areas of central Europe (Laibner, 2000). Its non-response to canopy openness is also surprising because *A. zebei* is known for its preference for shaded coniferous woodlands. On the other hand, it showed a negative relationship with plant cover at the understory, which could be the result of more opened canopy cover.

*Athous subfuscus* seemed to prefer sun-exposed sites in spruce dominated stands with mosses and shrubs at the understory level. Its high level of abundance and preference for spruce dominated stands correspond with recent data (Laibner, 2000; Kula, 2010). 

*Athous haemorrhoidalis* was not associated with any patch parameter other than the main tree species in species composition. This may be considered surprising, however, since it can also be frequently found in agricultural landscapes (Laibner, 2000).

## Conclusions

To the best of our knowledge, this is the first statistical evidence of the soil click beetle requirements within plantation forests, which provides some new or contrasting results with respect to the published evidence of distribution of these soil-dwelling taxa.

Some species from the genus *Athous* may reach relatively high levels of abundance in mature commercial stands and also those with a high proportion of non-indigenous Norway spruce plantations. Even though they are good dispersers, their response to the environment was over a relatively short patch radius and rapidly decreased with increasing study patch area.

Most of the studied species are rather beneficial organisms. Some of their larvae are predaceous on pests, which may contribute to the higher stability of Norway spruce plantations. Necrophagous larvae may contribute to the process of bioturbation, which is beneficial for nutrition availability or seed regeneration of the mature stands studied (e.g. Scheu, 1987). Thus, their levels of high abundance, and probable higher resistance to anthropogenic forest alterations, seem to be beneficial for commercially harvested woodland landscapes.

## Supplemental Information

10.7717/peerj.1568/supp-1 Supplemental Information 1 Raw data.

## Additional Information and Declarations

### Data Deposition

The following information was supplied regarding data availability: The raw data is supplied in the Supplemental Information.
