# Supplementary material for: The influence of mature oak stands and spruce plantations on soil-dwelling click beetles in lowland plantation forests
Source: PeerJ. 2016 Jan 12;4:e1568. doi: 10.7717/peerj.1568 (PMC4715454; doi:10.7717/peerj.1568)
Supplement: Supplemental Information 1 [file peerj-04-1568-s001.docx]

Species data:

|  | 1 | 2 | 3 | 4 | 5 | 6 | 7 | 8 | 9 | 10 | 11 | 12 | 13 | 14 | 15 | 16 | 17 | 18 | 19 | 20 | 21 | 22 | 23 | 24 | 25 | 26 | 27 | 28 | 29 | 30 |
| --- | --- | --- | --- | --- | --- | --- | --- | --- | --- | --- | --- | --- | --- | --- | --- | --- | --- | --- | --- | --- | --- | --- | --- | --- | --- | --- | --- | --- | --- | --- |
| haemorrhoidalis | 2 | 14 | 6 | 12 | 23 | 4 | 15 | 0 | 16 | 0 | 16 | 0 | 12 | 0 | 3 | 0 | 0 | 0 | 4 | 2 | 8 | 2 | 4 | 1 | 2 | 1 | 20 | 0 | 3 | 42 |
| subfuscus | 52 | 15 | 31 | 7 | 25 | 33 | 33 | 46 | 21 | 58 | 24 | 71 | 23 | 62 | 2 | 23 | 38 | 11 | 17 | 110 | 2 | 67 | 2 | 30 | 14 | 64 | 34 | 58 | 67 | 28 |
| vittatus | 0 | 0 | 0 | 0 | 0 | 0 | 0 | 0 | 1 | 0 | 0 | 0 | 0 | 0 | 12 | 0 | 0 | 0 | 1 | 0 | 2 | 0 | 0 | 0 | 0 | 0 | 0 | 0 | 0 | 5 |
| zebei | 22 | 2 | 12 | 2 | 5 | 23 | 3 | 1 | 3 | 5 | 3 | 10 | 4 | 16 | 0 | 11 | 11 | 19 | 4 | 14 | 2 | 4 | 0 | 29 | 0 | 8 | 0 | 29 | 15 | 0 |

Environmental data:

| 10 | 1 | 2 | 3 | 4 | 5 | 6 | 7 | 8 | 9 | 10 | 11 | 12 | 13 | 14 | 15 | 16 | 17 | 18 | 19 | 20 | 21 | 22 | 23 | 24 | 25 | 26 | 27 | 28 | 29 | 30 |
| --- | --- | --- | --- | --- | --- | --- | --- | --- | --- | --- | --- | --- | --- | --- | --- | --- | --- | --- | --- | --- | --- | --- | --- | --- | --- | --- | --- | --- | --- | --- |
|  | 1 | 2 | 3 | 4 | 5 | 6 | 7 | 8 | 9 | 10 | 11 | 12 | 13 | 14 | 15 | 16 | 17 | 18 | 19 | 20 | 21 | 22 | 23 | 24 | 25 | 26 | 27 | 28 | 29 | 30 |
| Shrub | 0 | 0 | 0 | 3 | 0 | 0 | 0 | 0 | 0 | 5 | 5 | 0 | 0 | 5 | 0 | 0 | 5 | 0 | 0 | 0 | 0 | 40 | 0 | 0 | 0 | 45 | 0 | 0 | 15 | 0 |
| Plant | 10 | 100 | 10 | 95 | 98 | 0 | 40 | 45 | 10 | 10 | 65 | 25 | 30 | 15 | 100 | 0 | 25 | 50 | 100 | 5 | 100 | 100 | 70 | 0 | 80 | 95 | 0 | 5 | 0 | 55 |
| Mosses | 60 | 0 | 0 | 0 | 0 | 10 | 0 | 50 | 5 | 5 | 5 | 0 | 0 | 20 | 0 | 5 | 5 | 0 | 0 | 30 | 0 | 0 | 0 | 0 | 0 | 0 | 0 | 5 | 60 | 0 |
| DB | 5 | 100 | 0 | 100 | 98 | 0 | 100 | 0 | 100 | 0 | 100 | 0 | 60 | 5 | 55 | 0 | 5 | 95 | 90 | 0 | 70 | 0 | 80 | 0 | 100 | 0 | 85 | 10 | 0 | 80 |
| SM | 75 | 0 | 100 | 0 | 0 | 100 | 0 | 80 | 0 | 60 | 0 | 80 | 0 | 50 | 0 | 100 | 60 | 5 | 5 | 100 | 0 | 80 | 0 | 90 | 0 | 100 | 0 | 85 | 95 | 0 |
| CO | 12.34 | 9.75 | 7.8 | 9.64 | 8.16 | 8.73 | 9.18 | 10.46 | 9.68 | 10.98 | 9.57 | 11.97 | 7.01 | 7.68 | 6.74 | 9.24 | 11.73 | 8.5 | 7.49 | 9.69 | 8.87 | 8.68 | 7.04 | 7.19 | 10.5 | 14.05 | 8.36 | 8.61 | 14.46 | 8.99 |

| 20 | 1 | 2 | 3 | 4 | 5 | 6 | 7 | 8 | 9 | 10 | 11 | 12 | 13 | 14 | 15 | 16 | 17 | 18 | 19 | 20 | 21 | 22 | 23 | 24 | 25 | 26 | 27 | 28 | 29 | 30 |
| --- | --- | --- | --- | --- | --- | --- | --- | --- | --- | --- | --- | --- | --- | --- | --- | --- | --- | --- | --- | --- | --- | --- | --- | --- | --- | --- | --- | --- | --- | --- |
| Shrub | 0 | 0 | 5 | 2 | 2 | 0 | 0 | 0 | 0 | 5 | 5 | 5 | 0 | 15 | 5 | 0 | 15 | 0 | 0 | 0 | 0 | 40 | 0 | 0 | 0 | 40 | 0 | 5 | 10 | 0 |
| Plant | 10 | 80 | 30 | 98 | 98 | 5 | 40 | 60 | 25 | 20 | 65 | 40 | 20 | 25 | 100 | 5 | 40 | 50 | 100 | 3 | 100 | 95 | 50 | 5 | 50 | 98 | 0 | 25 | 15 | 60 |
| Mosses | 60 | 0 | 0 | 0 | 0 | 5 | 0 | 35 | 5 | 5 | 5 | 0 | 0 | 20 | 0 | 3 | 5 | 0 | 0 | 40 | 0 | 0 | 0 | 0 | 0 | 0 | 0 | 10 | 50 | 0 |
| DB | 5 | 80 | 15 | 95 | 98 | 5 | 85 | 0 | 95 | 2 | 90 | 0 | 50 | 10 | 70 | 0 | 10 | 80 | 90 | 2 | 70 | 0 | 80 | 0 | 95 | 0 | 80 | 5 | 0 | 85 |
| SM | 80 | 5 | 80 | 0 | 0 | 90 | 5 | 50 | 0 | 60 | 10 | 50 | 0 | 60 | 0 | 100 | 55 | 10 | 3 | 93 | 0 | 80 | 0 | 80 | 0 | 90 | 0 | 80 | 80 | 5 |
| CO | 12.34 | 9.75 | 7.8 | 9.64 | 8.16 | 8.73 | 9.18 | 10.46 | 9.68 | 10.98 | 9.57 | 11.97 | 7.01 | 7.68 | 6.74 | 9.24 | 11.73 | 8.5 | 7.49 | 9.69 | 8.87 | 8.68 | 7.04 | 7.19 | 10.5 | 14.05 | 8.36 | 8.61 | 14.46 | 8.99 |

| 40 | 1 | 2 | 3 | 4 | 5 | 6 | 7 | 8 | 9 | 10 | 11 | 12 | 13 | 14 | 15 | 16 | 17 | 18 | 19 | 20 | 21 | 22 | 23 | 24 | 25 | 26 | 27 | 28 | 29 | 30 |
| --- | --- | --- | --- | --- | --- | --- | --- | --- | --- | --- | --- | --- | --- | --- | --- | --- | --- | --- | --- | --- | --- | --- | --- | --- | --- | --- | --- | --- | --- | --- |
| Shrub | 0 | 0 | 2 | 10 | 1 | 5 | 0 | 0 | 0 | 3 | 2 | 2 | 0 | 5 | 2 | 5 | 25 | 5 | 0 | 0 | 0 | 35 | 0 | 0 | 5 | 40 | 0 | 5 | 20 | 0 |
| Plant | 15 | 60 | 40 | 100 | 99 | 10 | 40 | 20 | 35 | 10 | 80 | 40 | 30 | 15 | 95 | 20 | 55 | 30 | 80 | 15 | 100 | 95 | 50 | 20 | 60 | 95 | 25 | 35 | 40 | 40 |
| Mosses | 60 | 5 | 0 | 0 | 0 | 10 | 0 | 75 | 2 | 5 | 5 | 0 | 0 | 10 | 0 | 5 | 10 | 0 | 20 | 40 | 0 | 0 | 0 | 2 | 0 | 0 | 0 | 5 | 30 | 0 |
| DB | 5 | 85 | 15 | 90 | 95 | 15 | 70 | 0 | 55 | 2 | 80 | 0 | 50 | 10 | 70 | 0 | 10 | 70 | 65 | 1 | 65 | 3 | 85 | 2 | 90 | 0 | 55 | 5 | 5 | 65 |
| SM | 80 | 10 | 75 | 7 | 1 | 75 | 20 | 50 | 20 | 60 | 10 | 50 | 0 | 60 | 0 | 85 | 45 | 15 | 25 | 60 | 0 | 80 | 2 | 60 | 0 | 90 | 0 | 90 | 70 | 25 |
| CO | 12.34 | 9.75 | 7.8 | 9.64 | 8.16 | 8.73 | 9.18 | 10.46 | 9.68 | 10.98 | 9.57 | 11.97 | 7.01 | 7.68 | 6.74 | 9.24 | 11.73 | 8.5 | 7.49 | 9.69 | 8.87 | 8.68 | 7.04 | 7.19 | 10.5 | 14.05 | 8.36 | 8.61 | 14.46 | 8.99 |

Coordinates:

|  | 1 | 2 | 3 | 4 | 5 | 6 | 7 | 8 | 9 | 10 | 11 | 12 | 13 | 14 | 15 | 16 | 17 | 18 | 19 | 20 | 21 | 22 | 23 | 24 | 25 | 26 | 27 | 28 | 29 | 30 |
| --- | --- | --- | --- | --- | --- | --- | --- | --- | --- | --- | --- | --- | --- | --- | --- | --- | --- | --- | --- | --- | --- | --- | --- | --- | --- | --- | --- | --- | --- | --- |
| x | 50.005 | 50 | 50 | 50 | 50 | 50 | 50 | 50.01 | 50 | 50.01 | 50 | 50.01 | 50 | 50 | 50 | 50 | 50.02 | 50 | 50 | 50 | 50 | 50 | 50 | 50 | 50.1 | 50.05 | 50 | 50 | 50.02 | 50 |
| y | 16.171 | 16.2 | 16 | 16.2 | 16.2 | 16.2 | 16.2 | 16.19 | 16.2 | 16.17 | 16.2 | 16.16 | 16.1 | 16.1 | 16.1 | 16.1 | 16.1 | 16 | 16.2 | 16.1 | 16.1 | 16.1 | 16.1 | 16.1 | 16.1 | 16.11 | 16.1 | 16.1 | 16.07 | 16.1 |
| xy | 808.62 | 809 | 810 | 810 | 810 | 810 | 809 | 809.4 | 809 | 808.5 | 808 | 808 | 807 | 807 | 806 | 806 | 805.1 | 804 | 808 | 808 | 808 | 808 | 807 | 807 | 807 | 806.6 | 804 | 804 | 803.9 | 804 |
| x2 | 2500.5 | 2501 | ### | 2499 | 2500 | 2500 | 2500 | 2501 | 2501 | 2501 | 2501 | 2501 | 2501 | 2501 | 2501 | 2501 | 2502 | ### | 2502 | 2502 | 2504 | 2504 | 2503 | 2503 | 2505 | 2505 | 2504 | 2505 | 2502 | 2502 |
| y2 | 261.5 | 262 | 262 | 263 | 262 | 262 | 262 | 262 | 261 | 261.3 | 261 | 261.1 | 260 | 260 | 260 | 259 | 259.1 | 259 | 261 | 261 | 260 | 261 | 260 | 260 | 260 | 259.7 | 258 | 258 | 258.3 | 258 |
